# Quality criteria and generalization of results from qualitative studies

**DOI:** 10.3402/qhw.v8i0.20647

**Published:** 2013-03-06

**Authors:** Lillemor Hallberg

A fundamental aim of all qualitative studies is to take the perspective of others and therewith to experience meanings, situations and actions through the eyes of others. This demands data from relatively small strategically selected study groups, in addition to empathy, openness and sensitivity in the researchers. Most qualitative researchers strive to come further than just pure descriptions in their analyses – they want to gain an in-depth understanding and explore meanings and processes of everyday life. Interpretation is therefore a basic tool included in all qualitative research.

There are many different qualitative methods – both descriptive and theory-generating. It is important that the specific research question guides the choice of qualitative research method rather than the other way around. The theoretical/philosophical basis for choosing a method should be shortly declared in a scientific article. Researchers also have the obligation to account for methodological details concerning for example how participants were selected and how variation in the data was secured; which type of data were collected, and how the analysis was carried through. Such information is necessary for the readers to be able to judge the quality and trustworthiness of the study and how reasonable the results are. The researcher is the “instrument” in a qualitative study and therefore he/she must account for personal as well as professional experiences that may affect data collection, analysis and interpretation of data. Ethical considerations are also important to account for in a research study on human beings. The ethical considerations include for example how participants were selected and informed about the study and in what way they gave their informed consent. In Sweden, we have a law (SFS 2003:460) concerning ethical policies in research on human beings conducted in Sweden, and most countries have similar laws.

Patton (2002) says that there is no burden of proof in qualitative research, and that it suffices if the world can experience and understand what the specific results tell the readers. It might be sufficient, but in my view this is hardly enough. My opinion is that all research, irrespective of method, should meet the same criteria of quality, validity and trustworthiness. Such criteria are presently expressed in different ways, for example as fit, work, relevance and modifiability (Glaser & Strauss, 1967). “Fit” means that the generated categories/concepts must be grounded in, and thereby fit, the data they represent. To reach such a fit demands that the variation in data must be fully captured. This requires a sufficiently large strategically selected sample. “Work” means that the result in a simple and obvious way describes or explains what is going on in the studied area. “Relevance” means that the study captures the readers so they recognize the studied reality in the results. The study, as well as its results, should also represent an area of real concern to people and not be of academic interest only. “Modifiability” means that the results of the study are not definitive and static – which implies that a changing reality will change the result, too. Quality aspects have also been expressed by other authors as credibility, originality, resonance and usefulness.

Many researchers are unsure about the question of generalizing results from qualitative studies. Of course, generalization cannot be done in the same way as results based on quantitative methods. Quantitative studies are most often based on random samples from defined populations. However, the result of a qualitative study, based on in-depth information from a strategically selected study group, can be tested in other contexts in order to validate the result.

Is it possible to reproduce results from a qualitative study? Based on the same theoretical perspective, principles for sampling, types of data and procedures for analyzing data, it is reasonable to argue that the results will be the same - or at least similar - if another researcher tries to replicate a qualitative study. I also think that it is possible to transfer – or generalize – the result of a qualitative study to other similar groups fulfilling the same selection criteria and circumstances in other respects.

*Lillemor Hallberg*Chief Editor

